# Peritoneal carcinomatosis of unknown primary site may be an undiagnosed appendiceal adenocarcinoma. A case series

**DOI:** 10.1016/j.ijscr.2019.09.005

**Published:** 2019-09-18

**Authors:** Paul H. Sugarbaker

**Affiliations:** Program in Peritoneal Surface Malignancies, MedStar Washington Hospital Center, Washington, DC, USA

**Keywords:** Peritoneal metastases, Small bowel adenocarcinoma, Meckel’s diverticulum, Urachal adenocarcinoma, Cytoreductive srugery, HIPEC, Intraperitoneal chemotherapy, Case series

## Abstract

**Background:**

Peritoneal carcinomatosis is sometimes diagnosed by cytology or by biopsy, but routine radiologic and endoscopic workup may not reveal a primary site. This clinical condition is called adenocarcinoma of unknown primary site.

**Methods:**

The clinical findings of two patients with adenocarcinoma of unknown primary site were presented and the implications for management of similar patients were suggested.

**Results:**

The patient had the diagnosis of adenocarcinoma of unknown primary site made when cancer was discovered at the time of umbilical hernia repair or laparoscopy. Radiologic and endoscopic tests were not definitive in providing the primary site for the malignancy. Exploratory laparotomy was performed which revealed an appendiceal mucinous adenocarcinoma as the cause of the peritoneal carcinomatosis. Cytoreductive surgery and hyperthermic intraperitoneal chemotherapy (HIPEC) were used at the time of the exploratory laparotomy to treat the appendiceal adenocarcinoma with peritoneal metastases.

**Conclusions:**

Peritoneal carcinomatosis of unknown primary site may be caused by an occult appendiceal adenocarcinoma. This error in diagnosis may lead to suboptimal treatment. Surgical exploration to visualize a normal appendix should occur prior to making a definitive diagnosis of peritoneal carcinomatosis of unknown primary site.

## Highlights

• Peritoneal carcinomatosis from unknown primary site must be a diagnosis of exclusion.
• Peritoneal metastases in the absence of a primary site by definitive radiologic and endoscopic tests does occur.
• An occult appendiceal adenocarcinoma may be the cause of the peritoneal carcinomatosis.
• Visualization of a normal appendix by exploratory laparotomy or laparoscopy is a new requirement for the diagnosis.

## 1 Introduction

Most patients with an abdominal or pelvic malignancy have a diagnosis made using conventional diagnostic techniques of radiology or endoscopy. The primary tumor is identified and then definitive biopsy is performed. In some patients the first symptom or sign of malignancy may be peritoneal carcinomatosis. Usually, the primary cancer site is identified by radiologic or endoscopic procedures and definitive biopsy obtained. In some unusual patients peritoneal metastases are documented but the primary site for the disease cannot be determined. The ascites fluid or peritoneal nodules containing the cancer cells are documented establishing the carcinomatosis but the primary site of the process is not discovered by conventional diagnostic tests. This is then carcinomatosis of unknown primary site [1,2].

Data on these two patients was prospectively recorded and then retrospectively reviewed at an academic institution. This research work has been reported in line with the PROCESS criteria [3]. This study was registered as a case series on the www.researchregistry.com website with UIN 4989.

## 2 Patient presentation

### 2.1 Patient 1

In May 2014, a 35 year old male had a new onset umbilical hernia repaired. Approximately one liter of mucinous fluid drained when the peritoneal space was entered. Biopsy from the hernia sac showed mucinous adenocarcinoma. Immunohistochemistry suggested origin in the gastrointestinal tract. Upper and lower gastrointestinal endoscopy was negative. CT showed ascites. The patient was given a presumptive diagnosis of adenocarcinoma of unknown primary site. He was started on 5-fluorouracil, leucovorin and oxaliplatin (FOLFOX) chemotherapy and completed six cycles over 3 months [4].

In October 2014, CT showed a reduced volume of ascites. The patient was asymptomatic. He was taken to the operating room with plans for exploratory laparotomy with possible complete cytoreductive surgery and perioperative intraperitoneal chemotherapy with curative intent [5,6]. The prior umbilical repair site was resected through a long midline incision using traction sutures [7]. The abdomen and pelvis including the small bowel and its mesentery was layered by mucinous adenocarcinoma and the possibility of surgically clearing small bowel of cancer was absent [8]. In this situation of peritoneal cancer index near maximum with high grade invasive adenocarcinoma, the surgical goals change to palliation and the identification of a primary site for the cancer.

The right side of the abdomen was clearly exposed. The caecum and ascending colon including terminal ileum were reflected from left to right to clearly expose the retroperitoneum. By visual inspection the appendix was absent and thought to have been destroyed by adenocarcinoma. A stapler was used to divide the caecum just below the ileocaecal valve and this specimen was sent separately to pathology to look carefully for remnants of a primary appendiceal mucinous adenocarcinoma. Other palliative surgical procedures were a greater omentectomy and tumor debulking. A palliative intraperitoneal chemotherapy regimen of hyperthermic liposomal doxorubicin at 100 mg/m^2^ for 3 h was used [9].

Pathology specimens all showed metastatic well differentiated mucinous adenocarcinoma. The specimen of caecum and appendix showed a mucinous adenocarcinoma of the appendix with peritoneal metastases. It was considered by the pathologist as the primary site for the disease. Five lymph nodes within the specimen were negative for cancer.

The patient received additional FOLFOX chemotherapy. He is alive with disease in June of 2019.

### 2.2 Patient 2

In October 2014, a 53 year old male began complaining of increasingly severe mid-abdominal pain. A CT, MRI and then laparoscopy were performed. The laparoscopy showed widespread mucinous adenocarcinoma. No primary site for the cancer was seen at the time of laparoscopy. Biopsies showed atypical cells suspicious for a gastrointestinal malignancy. Colonoscopy and upper gastrointestinal endoscopy were negative.

In November 2014, the patient was started on FOLFOX chemotherapy and he received 7 cycles (4). CEA was reduced from 125 prior to chemotherapy and 8 after its completion. On June 22, 2015, a repeat CT scan was performed which showed a reduction in the peritoneal metastases. It was thought that there was a complete or near complete response to systemic chemotherapy. On July 21, 2015, the patient was taken to the operating room where he had an exploratory laparotomy through a long midline abdominal incision. A greater omentectomy, cholecystectomy, and right colon resection with anastomosis was performed. The abdominal exploration showed fibrosis of the undersurfaces of the right and left diaphragm, right paracolic sulcus and pelvis. The small bowel was traced from the area of the ligament of Treitz to the ileocaecal valve. No obvious tumor masses were present on the stomach or large bowel. In reflecting the right colon from right to left, a diffuse mass was noted in the region of the appendix. The right colon and its adjacent lymph nodes were widely resected.

Upon histopathological examination the resected greater omentum showed foci of acellular mucus. The right colon and terminal ileum contained a high grade mucinous adenocarcinoma involving the wall of the appendix and periappendiceal tissues. Sixty-one lymph nodes were present in the specimen and all were negative for cancer. A Meckel’s diverticulum present in the terminal ileum was resected as part of the right colon specimen.

The patient received hyperthermic intraperitoneal chemotherapy with doxorubicin 15 mg/m^2^, mitomycin C 15 mg/m^2^, and 5-fluorouracil 400 mg/m^2^ and leucovorin 20 mg/m^2^ intravenously (6). Postoperatively the patient received early postoperative intraperitoneal chemotherapy with 5-fluorouracil for 4 days at 600 mg/m^2^ (6).

The patient recovered without adverse events. He received an additional 5 cycles of FOLFOX chemotherapy. He is alive without evidence of disease in June of 2019.

## 3 Discussion

### 3.1 New criteria for diagnosis of carcinomatosis of unknown primary site

In the past a diagnosis of carcinomatosis of unknown primary site required a positive cytological biopsy, negative radiologic tests, usually an abdominopelvic CT, and negative upper and lower gastrointestinal endoscopy [1,2]. In this clinical situation a diagnosis of carcinomatosis of unknown primary site was made. In some patients immunohistochemistry was of help in establishing a diagnosis [2]. As a result of extensive experience with appendiceal adenocarcinoma, I suggest that this diagnosis not be used unless the patient has an exploratory laparotomy with complete visualization of the right colon, caecum, and appendiceal region. If a normal appendix is visualized and no other sites of occult adenocarcinoma are identified, then the diagnosis of carcinomatosis of unknown primary site is appropriate. Laparoscopy can be used to rule out an occult appendiceal adenocarcinoma only if the entirety of the appendix is visualized.

### 3.2 Clinical characteristics of an occult appendiceal adenocarcinoma

There are several characteristic features of an appendiceal adenocarcinoma that result in great difficulties in diagnosis in the absence of exploratory laparotomy. Of course, an upper gastrointestinal endoscopy will be negative. Also, colonoscopy to the caecum is usually negative unless the adenocarcinoma is at the orifice of the appendix. For perforation and peritoneal seeding to occur, the adenocarcinoma is usually not at the appendiceal orifice but somewhere along the shaft of this structure. Here perforation would occur into the free peritoneal cavity with widespread dissemination of cancer cells into the peritoneal space. CT may be negative because of the small size of the primary tumor. An appendiceal adenocarcinoma, especially a mucinous malignancy, may cause extensive peritoneal metastases in the absence of a mass identified on CT. If the appendiceal primary adenocarcinoma is retrocaecal, especially if there is surrounding inflammation, laparoscopy may not be able to visualize the lesion. Finally, the occult primary appendiceal adenocarcinoma may not cause any symptoms and signs. The perforation of the appendix by cancer may occur in the absence of pain or localized tenderness typical of appendicitis. Bleeding from the primary cancer never seems to occur as is frequently observed in gastric cancer, small bowel adenocarcinoma, or colon cancer. Finally, because the malignancy is outside of the passage of enteric contents, bowel obstruction does not occur.

### 3.3 Other causes of an occult primary malignancy causing peritoneal metastases

Other unusual malignancies may present with peritoneal metastases and a negative diagnostic workup. Ectopic gastric or pancreatic epithelium in a Meckel’s diverticulum may present as peritoneal metastases [10]. Also, a urachal mucinous neoplasm may develop peritoneal metastases and the primary tumor not cause symptoms or signs [11]. Although small bowel adenocarcinoma may not be seen by endoscopy or imaged by radiology, it usually causes pain from obstruction or bleeding. In the absence of symptoms small bowel adenocarcinoma may result in adenocarcinoma of unknown primary site. Finally, malignant peritoneal metastases may occur and no primary site is present. Malignant peritoneal mesothelioma is a primary cancer of the peritoneum itself. If cytology is obtained or a biopsy of the peritoneal metastases is possible, the pathologist can perform the appropriate immunostains and identify the peritoneal metastases to be from malignant peritoneal mesothelioma [12].

### 3.4 Requirement for a treatment strategy for peritoneal metastases

The surgeon who is responsible for the exploratory surgery must be prepared to definitively treat the peritoneal metastases. Knowing that a definitive diagnosis of an appendiceal primary cancer may occur, an appropriate treatment strategy needs to be available prior to exploration. A new standard of care for appendiceal neoplasms with peritoneal metastases has been reported and is globally accepted [13]. Cytoreductive surgery combined with perioperative intraperitoneal chemotherapy should be used if any occult appendiceal malignancy can be identified. [5,6]. The patient must be made aware of the possibility of an extensive surgery that requires peritonectomy procedures and visceral resections. After the cytoreductive surgery is complete, hyperthermic intraperitoneal chemotherapy (HIPEC) is used. In order to properly execute treatments for peritoneal metastases, considerable experience and special training is necessary [14].

### 3.5 Implications of an appendix destroyed by adenocarcinoma

At the time of exploratory laparotomy, peritoneal carcinomatosis of unknown primary site should not be used as a diagnosis unless a normal appendix is visualized. The appendix cancer may not be obvious at the time of surgical exploration. A high grade appendiceal adenocarcinoma may totally destroy the appendix and peritoneal metastases occupy the anatomic site for the normal structure. Realizing this, the diagnosis of peritoneal carcinomatosis of unknown primary site is made only if a normal appendix is visualized. An absence of the appendix indicates appendiceal cancer that has destroyed the normal structure. This indicates an invasive process and a guarded prognosis may occur even with adequate treatment.

In order to gain maximal information regarding the primary site, the appendix or the caecum and appendix must be resected including as much surrounding tumor as possible. Sometimes a right colon resection is indicated. The tissue specimen that contains a possible occult appendiceal malignancy must be oriented for the pathologist with specific instructions that there is a high index of suspicion that the cancerous mass may contain the primary cancer. If the tissue from the anatomic site of the appendix and caecum is sent along with a large cancerous mass from greater omentum and areas of tumor debulking, the occult appendix cancer is most likely to be missed by pathologic examination.

## 4 Conclusions

In order to optimize the treatment of cancer patients, an accurate diagnosis of the site of primary disease is necessary. The diagnosis of peritoneal carcinomatosis of unknown primary site is a diagnosis of exclusion. All possible primary sites for the disease need to be ruled out. An occult appendiceal adenocarcinoma may perforate and seed the abdomen with peritoneal metastases. An absence of symptoms and an absence of definitive radiologic and endoscopic tests may exist with appendiceal adenocarcinoma. An exploratory laparotomy and visualization of a normal appendix is a requirement to preserve a diagnosis of peritoneal carcinomatosis of unknown primary site.

## Funding

Data management and secretarial support provided by Foundation for Applied Research in Gastrointestinal Oncology.

## Ethical approval

Local IRB-approval for this case report was not required:

MedStar Health Institutional Review Board has determined that a case report of less than three (3) patients does not meet the DHHS definition of research (45 CFR 46.102(d)(pre-2018)/45 CFR 46.102(l)(1/19/2017)) or the FDA definition of clinical investigation (21 CFR 46.102(c)) and therefore are not subject to IRB review requirements and do not require IRB approval.

This case series is of 2 patients.

## Consent

Written and signed consent was obtained from the patients.

## Author’s contribution

Paul H. Sugarbaker, MD: study concept or design, data collection, data analysis or interpretation, writing the paper.

## Registration of research studies

This study was registered as a case series on the www.researchregistry.com website with UIN 4989.

## Guarantor

Paul H. Sugarbaker, MD.

## Provenance and peer review

Not commissioned, externally peer-reviewed.

## Declaration of Competing Interest

Paul H. Sugarbaker has no conflicts of interest to declare.
